# Spirit of the British and American Periodicals, &c.

**Published:** 1842-10-01

**Authors:** 


### Spirit of the British and American Periodicals

### MESMERO-PHRENOLOGY.

A MARRIAGE extraordinary has lately taken place between phrenology and mesmerism, to the great scandal and indignation of the rational and sober advocates of the former science. If Mother Mesmer prove as prolific of her after-births as she has been in this her first litter, we shall soon have such a swarm of hybrid monsters as would have frightened Deucalion and Pyrrha out of their seven senses.

> Terruit Gentes grave ne rediret
> Sæculum Pyrrhæ, NOVA MONSTRA.—

We shall exhibit one or two specimens of the unhallowed brood which this unnatural alliance has already given birth to. The profession may well be characterised as in a state of anarchy—or rather insanity, when such examples of mental obliquity, infantile credulity, and astounding self-delusion are exhibited to the world!

Mr. Brookes, of Liverpool, was the accoucheur who brought into this world the following sample of the first-born of the matrimonial alliance under consideration.

Sarah Badger, from her 14th year, was subject to epileptic fits—and from 14 to 17, was seldom many days without them. She ultimately became insane, and was confined in more than one work-house or asylum. She was therefore an excellent subject for giving birth to mesmero-phrenological bantlings—at least according to Mahomedan belief.

After one of her releases from lunatic restraint, Mr. Brookes took her into his service, but the fits became more decided, and the insanity returned. Before she could be got into another asylum various mesmero-phrenological experiments were made on certain of her organs—especially TIME *and* TUNE, which we must pass over. After another year's incarceration, she again came into the service of Mr. Brookes, and it is at this juncture that we shall quote the words of the narrator.

"In November last I began to mesmerise her, and she soon became lucid. On the 16th of December, *she directed me* to mesmerise her on the 20th, and permit her to sleep for 24 hours, during the last of which she said she should be able to tell us the cause of her fits, if they were curable, and what remedies should be applied. She was laid to sleep accordingly, and about the tenth hour there appeared symptoms of considerable determination of blood to the head, and she began to hear and speak of the most exquisite music, and asked who was playing, and where, &c.? By and by she began to sing to it, at first feebly, but after the first minute or two with her usual power.

"The physical exertion, I had no doubt, would be injurious, as she was in a very exhausted state; and I therefore endeavoured to restrain it. With this view, I breathed strongly on the organs of *Time* and *Tune*, and she gradually ceased singing, saying, 'They play it very badly—I can hardly hear them now—humph, they've stopped now.' In a minute or two after I had ceased breathing upon her, she said, 'Hush! now they're playing—

'Gaily the Troubadour,'

and immediately began to sing it—starting about the middle of the tune—not at the commencement. *I then pointed my fingers to her mouth, rendered her tongue*

*cataleptic, and sealed her lips up;* but she still continued the efforts to sing, producing the usual humming sounds through the nostrils for a short time, and then ceased. On releasing her mouth, I inquired, 'Is the music playing now?' 'Yes,' she replied, 'very prettily.' 'Then, why did you stop singing?' 'I could not help it—I could not sing.' 'Why?' 'Because you forbid it.'

"She still heard the music several times after this, and joined it with her voice; but as often as she did so I renewed my operations on *Time* and *Tune*, and stopped it. At length the frequent stoppings annoyed her, and she said, 'They played so very badly, and stopped so often, she would listen to them no longer.'

"On the 28th of December, after considerable exertion in the somnambulic state, she appeared much fatigued, then became very happy, and heard sweet music—*a violin.* A large *musical box* was placed close to her ear whilst playing a *lively waltz.* 'Do you hear the music now?' 'Yes.' 'What is it?' '*A violin.*' 'What is it playing?' '*Home, sweet home.*' I breathed upon *Time* and *Tune*, and she said, 'the music had *stopped.*' The musical box was *still playing.*

"Several ladies were present, who wished to hear her sing, and she was permitted to sing several songs. Whilst she was singing I again cataleptised the *tongue and jaws,* and sealed the *lips up and she continued.* I again breathed on the organs, and stopped her in the middle of the tune, and she said, she stopped 'because the music stopped—she did not know why.'

"On being awoke soon after, she complained of pain in the forehead. 'Where is it—here?' said Mr. D., placing his finger on the middle of her brow. 'No, not there—on the side.' She was directed to place her finger on the spot, and she raised her hand, and placed her finger precisely on the organ of *Tune.* A short time after Mr. D. said, 'Did you say this was the spot where you felt the pain?'—putting his finger on the organ, when she instantly shrunk from it as if it gave her great pain, saying 'It was so very sore just there, she could not bear it touched.'

"Other parts of the forehead were touched, and it did not give her pain, only at the seats of *Time* and *Tune*; and she said she had not felt the soreness before, and did not know the cause, 'unless we had been doing something to her during her sleep that had caused it.'

"On a previous occasion, whilst walking about in the somnambulic state, she was much distressed, and complained that I was leading her down a fearful 'steep hill,' and was immediately relieved of her apprehensions by my breathing on the organ of *Weight.*

"Though I had carefully noted the foregoing acts, as confirmatory of the organs in which they spontaneously exhibited themselves, yet it never occurred to me to attempt exciting any of the other organs until Saturday the 15th ult. when I received an old 'Liverpool Albion,' containing a report of the experiments of Drs. Buchanan and Collyer, and then of course felt surprised the experiments had NOT occurred to me before.

"I proceeded forthwith to test them—my first operation was on *Benevolence,* and was much surprised to find it produced effects directly contrary to those I expected: in fact, decided manifestations of *Combativeness* and *Destructiveness,* which for a time rather perplexed me, so I next tried the two latter organs, and found the manifestations totally changed. I next tried *Cautiousness,* and produced *Fear* and *Despondency,* and my operations on *Hope* and *Ideality* seemed only to increase her misery; but on my again operating on *Cautiousness,* she became better, and a further operation on *Hope* and *Ideality* made her perfectly happy and cheerful. From these results I of course inferred that by one mode of operating, the organ might be stimulated to greater activity, and by another mode might be rendered incapable of its ordinary activity.

"On a subsequent occasion, I had had my hand on her forehead, for the purpose of assisting her in examining the brain, and then endeavoured to stimulate the whole of the intellectual organs, and the following were the manifestations—

'O, dear, I feel so well and so different; I feel as if I should like to sit and talk for hours—O, I wish I could find words to express myself—I don't know how I feel hardly; but so happy, and clear, and light-hearted—and—and *affectionate like, (Benevolence.)* And what's the cause of all this?'

" 'I really don't know—I feel quite a different thing—I could *discuss* with anybody now—on any subject—I feel capable of anything—and of talking to anybody—O, if I could but find words to express myself—I think my head must have some of yours about it, somehow, for it has more in it than ever I knew before—I'm sure I'm *cleverer* now than I used to be—do converse with me about something—any subject—what *shall* we talk about,' &c.

"I have since on several occasions, tried other organs singly and with similar success—generally producing increased activity, but sometimes rendering them somnolent, and leaving their antagonist's faculties in unrestrained activity."*

If the worthy Lord Mayor of Dublin should peruse the above narrative, he would be apt to exclaim—"this bangs Bannagher." Observe the beautiful simplicity and truth with which the Prophetess delivers her oracular responses. She desired Mr. Brookes to lay her asleep for 24 hours, in order that she might tell the nature of her fits, and the proper remedies. In due time she developed these important matters by "hearing and speaking of most exquisite music!" There's a lecture of etiology and therapeutics for you!! Then a musical snuff-box was applied to her ear. She declared it was a *violin!* The tune it played was a "*lively waltz*," (in Mr. Brooke's apprehension at least) but the Prophetess asserted that it was "*Home, sweet home*"!!

Then again, the operator rendered the tongue of the Prophetess cataleptic, and sealed her lips up. Yet she *continued to sing* for the amusement of some ladies that were present! This shews how difficult it is to stop a lady's tongue, whether Mesmerized or not. The consistency and truth of Mesmero-phrenology are beautifully illustrated by certain experiments which Mr. Brookes made "on Saturday the 15th ult." of some month unnamed. His first operation was on the organ of *Benevolence,* when decided manifestations of *Combativeness* took place!!!

Let the rational phrenologists look to this in time. When we see the public marriage between Mesmerism and Phrenology, and the meretricious Harridan introduced into a phrenological institution as a modest woman, we apprehend that phrenology is in danger, and that there are "snakes in the grass." We have strong suspicions, indeed, that Mr. Brookes himself is a sly anti-phrenologist, as well as a secret enemy of Mesmerism, who, under the mask of disciple and true believer, has taken a most ingenious way of damning both the one and the other science.

Be this as it may, we shall now advert to a narrative, the author of which cannot be suspected of insincerity, however much we may consider him the victim of credulity and deception. It appears that a Capt. Valiant, of the 40th Regiment has a worthy and attached nurse, who, under the influence of magnetic Mesmerism, [communicated from Sir Thos. Wilsher *and Sir Thomas alone*], underwent a surgical operation while entranced by that gentleman, without knowing, or at least acknowledging, that the said operation was being performed! Crædat Judæus. This faithful nurse appears to have learnt her part to admiration. She has beaten Miss Okey out of the field. By the way, where is Miss Okey? Are her exhibitions *confined* to private rehearsals now? This nurse, then, came to Dr. Elliotson, at a time when Mr. Brookes was in the Doctor's library, with his patient Miss Badger, the heroine already noticed. The "short, delicate, artless woman, 30 years of age," (the nurse) offered to submit to the Doctor's manipulation, provided her mistress, who is, no doubt a true believer, were present.

---

* Medical Times, June 11, 1842.

After the Doctor had thrown the "artless woman," into profound mesmeric sleep, the following phenomena were elicited.

"On touching the organ of Self-esteem, she rose instantly from her seat, elevating her hand as high as possible, speaking with the utmost pride and disdain, displaying the natural language of these emotions in the most exquisite manner. On withdrawing my finger and placing it upon the organ of Benevolence, the change was instantaneous to a humble and kind expression of countenance and voice, and she was all gentleness and goodness. On placing my finger on the organ of Wit, she became at once most merry and facetious. On pointing over the organ of Parental Love and Attachment, she became serious and gentle, and the tears streamed down both cheeks; indeed, her distress was such that I was obliged to touch over some other organ. She talked of her husband, whom she had not long seen, and of Mrs. Valiant's baby. On touching over Destructiveness, her violence was outrageous; but instantly changed to pride, gentleness, melancholy attachment, or wit, when my finger left it for the respective organs of those faculties. *I played upon her brain, by my fingers, as readily as upon a piano-forte.** Great as are the miracles of Mesmerism, witnessed by me now for above five years, these phenomena surpassed all."†

*Eheu jam satis.* Dr. Elliotson feelingly and somewhat indignantly deplores the indifference which the profession has shewn to the miracles of Mesmerism. He says they are as indifferent to Mesmeric facts, "as the cattle grazing in the meadows are to the wonders of the steam-carriages passing by them on the railroads." The simile is more applicable than Dr. Elliotson supposes. When the trains first began to run, the cattle scoured away in all directions, as if some DEMONS OR SUPERNATURAL BEINGS were thundering along the road. But TIME generally removes the film from our mental as well as our material optics —and this seems to have been the case with the cattle, who soon found out that there were no GIANTS OR HOBGOBLINS in the trains—and probably the bipeds will ultimately discover that there is neither magic nor magnetism in the Mesmeric passes, but a precious lot of humbug, credulity, and delusion.

---

### CARTILAGINOUS DISEASE WITH PERFORATION OF THE STOMACH. By Dr. KENNION.‡

On Friday, June 10th, Dr. Kennion was requested to see Mr. T. æt. 49, in consultation with Mr. Parry. He was somewhat emaciated, his countenance anxious; pulse 76, languid; tongue white and swollen. His principal complaint was of extreme distention of the bowels, "as if he would burst;" there was no tenderness on pressure; the motions were said to be black and offensive. His appetite was tolerably good, and he never suffered any inconvenience after eating beyond that arising from the flatulence.

It appeared that the patient had been out of health for several years, but had become much worse within the previous six months; the principal symptom has been the frequent recurrence of paroxysms of intense pain, generally coming on at night, and referred almost entirely to the umbilical and hypogastric regions; these paroxysms were always connected with a great degree of flatulent distention, and generally passed off with the discharge of flatus.

Under these circumstances he came to Harrogate, but without at first deriving much benefit. The treatment adopted, consisted of small doses of blue-pill,

---

* We should like to know which of the two predominates in the Mesmero-Phrenological head—*Piano* of the brain, or *Forte* of the skull?

† Medical Times, 25th June, 1842.

‡ Prov. Med. Journ. July 2.

extr. of colocynth and hyoscyamus to regulate the bowels; the nitro-muriatic acid, with decoction of taraxacum as a general tonic and with the view of acting upon the liver, which was evidently in fault; and the use of the assafœtida lavement, for the purpose of relieving the tympanitic state of the bowels; at the same time his diet was carefully regulated.

He appeared to improve under this line of treatment, when suddenly he was seized with the most agonizing pain over the whole abdomen, which was so tender that he could not bear the slightest touch; the pulse was extremely rapid, the countenance anxious. In a few hours he expired.

On opening the abdomen, there were found more than three quarts of turbid serum, with some flakes of lymph. The bowels were enormously distended. On the anterior border of the lesser curvature of the stomach, were found two irregular openings, of about three-quarters of an inch in diameter, the margins of which were as thin as a piece of paper. These perforations were about an inch and a half from the pylorus, and were situated in the centre of a mass of cartilaginous substance, which involved the whole circumference of the stomach, for a distance of from three to four inches from the pylorus. The mucous membrane lining this diseased portion did not present any unhealthy appearance, excepting that in one or two small patches it was slightly turgid and of an ashy hue. But the muscular coat seemed to have disappeared entirely, and to have been replaced by cartilage. The thickness of this substance varied from a quarter of an inch to an inch; it had a nodulated appearance, and when cut into was perfectly white, homogeneous in structure, and without any bands or striated appearance whatever. The pylorus itself was only slightly thickened from an hypertrophied state of the mucous membrane.

In some parts the serous coats of the stomach was involved in the cartilaginous degeneration, while in others it was quite healthy.

Attached to it in different places, by short pedicles, were a large number of small fatty tumors, each contained in a fibro-serous capsule.

The liver was congested; the gall-bladder filled by an enormous gall-stone. The rest of the abdominal viscera were perfectly healthy.

---

### Case of Venous Hæmorrhage from an Abscess. By Robert Storrs, Esq. Doncaster.*

The following case bears some analogy to that one of Mr. Liston's lately discussed at the Medico-Chirurgical Society.

William Heaton, a shoemaker, aged 36, was seized, Dec. 14, 1839, with severe febrile symptoms, accompanied by an extensively diffused swelling over the front of the throat, of a phlegmonous, erysipelatous character. In spite of leeches, fomentations, poultices, &c. suppuration commenced, and on the 21st evident fluctuation could be felt on the left side of the larynx. As the constitutional symptoms were very severe, the abscess was opened immediately, and above a pint of very offensive sanious pus was discharged, accompanied with some large shreds of what appeared to be then dead cellular structure. About a pint or more continued to be discharged daily, and the sac of the abscess was found to extend itself from the left of the larynx and trachea, to the nape of the neck on that side, and from the angle of the jaw to the clavicle.

On the 30th some hæmorrhage occurred from the opening, evidently of venous blood, which was controlled by pressure over the internal jugular; this pressure was kept up, and though the discharge of pus continued, no blood es-

* Provincial Medical and Surgical Journal, April 2, 1842.

caped until the night of the 1st of January, when it recurred and was again stopped. During the following night, however, it again burst forth, and on the following morning he died.

No examination of the body was permitted, but there could be no doubt that the abscess had implicated the internal jugular vein, the coats of which had been destroyed, and had probably been partly cast off at the time the abscess was opened, and that a large discharge of blood had been then prevented by the deposition of coagulable lymph at the venous orifice.

---

### CASE OF LARDACEOUS CANCER OF THE CRANIUM. By ALFRED BAKER, Esq.*

Joseph Rollason, æt. 31, a glass-cutter, of intemperate habits, states that about three years ago, being intoxicated, he fell and cut the forehead on the left side. This wound soon healed and he never experienced any ill effects from it. About seven months ago, after residing in a damp situation, he was attacked with violent pain in the head, occasional giddiness and dimness of sight. He also felt a dull and heavy pain in the left arm, with numbness and loss of power in the middle finger, and in a few days afterwards, pains in the lower extremities, especially in the joints. The pain increased, and was accompanied with a small and soft swelling over the part formerly cut. About eight weeks after the attack commenced, he complained of pain within the head, the previous pains being situated in the scalp and on the surface; he had also pain and stiffness in moving the jaw, and was only able to open his mouth to a small extent. In the course of a few days numbness and tingling down the left side of the face and left arm came on, and the sight, especially with the left eye, became affected. The swelling over the left frontal eminence, where the wound had been, began to enlarge without pain, and acquired considerable hardness.

He remained much in the same state for about four months, when suddenly the pain in the head was aggravated, and the tumor, which had been slowly enlarging, became tender to the touch. The tingling sensations were worse, and affected both sides of the face, but more especially the right. There was also puriform discharge from the nostrils, occasionally mixed with blood.

At this time, (April 10th), there is a large ovoid tumor on the left side of the frontal bone, of about two-thirds the size of a duck's egg; it was hard, lobular, and appeared to be intimately connected with the bone; the integuments are not affected. There is also another smaller swelling, soft though still firm, on the right side of the skull near the lambdoidal suture. The two sides of the face are not quite symmetrical, the left cheek being slightly drawn. The pupils are contracted and insensible to light; he is unable to discover even a lighted candle placed before him; the left eye is more prominent than the right. Powers of smelling and tasting considerably impaired, but hearing is not much affected. Ordinary sensation not affected in any part of the body, but the motory power of the left arm is much enfeebled. Sphincters not affected; mind slightly impaired; breathing slow and easy; pulse 112, weak and small, skin cool, bowels open. He is taking the bichloride of mercury. By the 4th of May, both eyes had become injected, and the cornea in each subsequently ulcerated.

May 19th. The patient has been progressively getting worse. He lies in a state of semi-coma, but capable of being roused, when he replies to questions, articulating his words imperfectly, and talking unconnectedly. The tumors on the head have each enlarged, and become firmer; ptosis of upper eyelids; both

---

* Provincial Medical Journal, July 16.

corneæ ulcerated; there is a hard swelling over the left malar bone, unattended with pain; his breath is extremely fetid, though he has taken no mercury for three weeks; respiration stertorous; some difficulty in deglutition; he continues to discharge a bloody matter from the nostrils; pulse 90, small and soft. Ammonia, wine and water, beef-tea.

May 27th. Has been occasionally delirious. Another tumor discovered over the right ascending ramus of the lower jaw; it is hard, ovoid, free from tenderness and connected with the bone. Has taken no food for the last two days, and the increased pressure on the brain has been marked by a proportionate coma, with stertor in breathing. He died at eleven o'clock this morning.

*Sectio Cadaveris.—Cranium.*—The scalp over the large tumor was very adherent; the tumor lay beneath the occipito-frontal aponeurosis and the pericranium, and was lobulated on its surface. The smaller swelling behind, was found to consist of a white medullary substance containing numerous red patches, as though from torn vessels and ecchymosis. The bone was seen at the bottom, and was eroded and cellular. When the calvarium was removed, the large anterior tumor was found to project inwards upon the brain, its inner portion not being so prominent as the external; it was enclosed in a dense whitish membrane, not adherent to the dura mater. On separating the calvarium, the tumor was seen clearly to be a growth within the proper tissues of the bone; on being divided it was found to consist of white lardaceous matter deposited within the pericranium. It was divided into two portions by the bone which traversed it nearly across the centre. The bone at this point appeared at first to be thicker than natural, but this was found to depend upon the deposition of the cancerous matter in the cells of the diploe, and upon a separation of the denser parts of the bone into laminæ which were divided from each other by white lines of the same material.

The brain itself was uniformly pale and bloodless, the cineritious structure being of faint yellowish-grey colour. Upon examining the base of the cranium, the sella turcica was found to be occupied by a tumor, which projected over the smaller wings of the sphenoid and fore-part of the petrous bone, involving and compressing the contents of the cavernous sinuses and the fifth nerves with the Casserian ganglia. This growth had the same characters as those already described, and the bones beneath it were similarly diseased. On the left side the bones of the orbit seemed to be more changed than on the right, and by their tumefaction to have protruded the globe of the eye.

*Abdomen.*—The liver was large, dark in colour, and contained three or four masses of medullary or lardaceous cancer, of pulpy consistence and white colour, with spots of deep red and softer quality, as though from the process of ramollissement, and effusion of blood in consequence of ruptured vessels. One of these was as large as the palm of the hand.

*Remarks.*—The numbness and tingling in the left arm occurred before any external swelling was perceived. This would lead to the opinion that the disease first protruded in the interior of the cranium. The numbness and tingling, first in the left cheek, and subsequently in the right, show the early commencement of that medullary growth which was found in the base of the cranium, the extent and connexion of which explain satisfactorily the altered sensations in the soft parts, the amaurosis, the discharge of blood and matter from the nose, the protrusion of the left eye, and the ptosis of the left upper eye-lid, for it involved the optic nerves, the third, fourth, fifth and sixth as they pass through the cavernous sinus, the pituitary body, and the sphenoidal and ethmoidal cells, as well as the bones of the orbit. The occurrence of opacity and ulceration of the corneæ indicated destruction of the fifth nerve, and was accompanied by a pale chemosis.

The difficulty of opening the mouth depended probably not upon any nervous lesion, but on the mechanical impediment to the motions of the jaw, caused by the tumor on the ascending plate of the lower maxilla on the right side.

Lastly, with regard to the nature of the disease, Mr. Baker regards it as white medullary or lardaceous cancer of the bones of the cranium, and the characters of the tumors in the liver, in some measure strengthen this view.

### FRACTURE OF THE FORE-ARM; GANGRENE PRODUCED BY THE APPLICATION OF THE STARCHED BANDAGE.

In speaking of fractures of the middle part of the fore-arm, M. Roux recommends that the bandage should not be too tightly applied, as the principal veins of the extremity being superficial, they are easily compressed; venous circulation is impeded and gangrene may ensue. For this reason he never employs in these cases moistened bandages, or starch or gum splints. A case came in to the Hôtel Dieu in 1841, which completely realized these fears; a moistened apparatus had been employed on the fore-arm of a child; gangrene supervened, and it was necessary to have recourse to amputation.

*Case.*—A child, æt. 12, of delicate constitution, fractured both bones of the fore-arm by a fall from a carriage; the surgeon applied the ordinary apparatus, which he had previously moistened with clean water; this tightened application caused horrible sufferings during four days, when the surgeon replaced it by the starched bandage, which was less tight than the first, so that it was more easily borne; nevertheless, two days afterwards, dark vesications showed themselves at the extremities of the fingers, and on the 12th the patient was brought to the Hôtel Dieu, with well-marked gangrene of the hand and lower part of the fore-arm. On the following day amputation of the fore-arm was performed. The patient ultimately recovered.—*Gazette Medicale de Paris.*

### CASE OF AMPUTATION OF THE LEG, WITH SOME OBSERVATIONS ON A NEW MODE OF AMPUTATING. By THOMAS GREEN, Esq.*

James Allpass, æt. 34, a butcher, was admitted into St. Peter's Hospital, in 1842, under Mr. Green.

About four years ago the patient had a severe compound fracture of the right leg; the bone united, but obliquely, and there is now considerable projection of bone, with extensive ulceration of the skin around. On admission the ulcer was in a sloughing condition, with much sanious discharge, the surrounding skin was of a fiery red colour, and the whole extremely painful; the limb shortened, so that he cannot bring the heel to the ground.

It was considered necessary that the leg should be removed, which was done as soon as the patient was in a fit state. Mr. Green decided on performing the flap operation, but in a different manner from that in which it is usually done. A transverse incision having been made across the front of the leg, through the skin, another was made through the integument at the back of the limb, including a large portion of the calf, and leaving skin enough to cover the flap of muscle, which was next formed by passing the catlin through the leg a short distance behind the bones, and cutting out in the usual way; the remaining muscles were divided by a transverse incision passing between the bones, which were next sawn through and the arteries tied. Three sutures, and pieces of strapping were applied to keep the posterior flap in apposition.

---

* Provincial Medical Journal, July 2, 1842.

Three days afterwards, secondary hæmorrhage occurred, which was at last arrested with some difficulty. In six weeks the stump was healed and in good condition.

Mr. Green afterwards made some remarks upon the method of operating which he had had recourse to. In amputating below the knee, he said, he usually employed the flap operation, but several disadvantages attended this operation as usually performed, one of these was the projection of the muscle beyond the skin in muscular persons; a second was, the oblique division of the nerves: and, lastly, the oblique division of the vessels. To obviate these disadvantages many plans had been adopted. The mode which Mr. Green pursued was as follows. An incision was made anteriorly across the fore-part of the leg, in the usual situation, about two inches below the tuberosity of the tibia, extending from the inner angle of that bone to a point behind the fibula; from the termination of this incision, on the inner side of the leg, the knife was carried downwards to some extent, next across the limb posteriorly in a curved line, and brought up at the outer side, so as to unite with the front incision behind the fibula. In this manner a portion of integument was divided, which might be correctly described as representing two-thirds of an oval figure.

This incision should go through the skin and subjacent tissue, down to the fascia covering the muscles; the contraction of the integument itself, with a trifling assistance by drawing the skin upwards, leaving a separation of about half an inch between the edges of the incision. A long catlin was now pushed through the leg, about one-third of an inch behind the bones, and carried downwards and next backwards, so as to make a flap of muscle, its edges corresponding with those of the retracted integuments. The remaining muscles were next divided transversely; in this division are contained the large vessels and nerves, which are those cut transversely. The bones were separated, the sharp angle of the tibia was sawn off, and the arteries secured in the usual way. The flap, when brought up over the face of the stump, was entirely and abundantly covered by skin: three sutures were used, assisted by two broad pieces of strapping. These are to be removed on the third day.

By performing the operation in this manner, Mr. Green says, you will usually secure a good stump.

---

### On Benzoic Acid in Urinary Disorders. By John Smith Soden, Esq.*

Mr. Soden's attention having been drawn to the efficacy of benzoic acid in certain disordered states of the urinary organs, he was induced to give this medicine a trial at the Bath United Hospital; the following are the heads of four cases in which it was used in that institution.

*Case* 1.—A man, aged thirty-five, complained of frequent desire to make water, which had existed for a month; the urine deposited a mucous sediment. On passing a catheter the urine was found to be natural, but there was some hæmorrhage after the urine had been drawn off; some pain in the loins. Was first cupped on the loins, and aperients ordered; and then diosma and pareira brava, with opiates, were given in succession. At the end of three weeks, the bladder remained in the same state. He was then ordered to take the mixture, with benzoic acid and balsam of copaiba. In two days he was much better, in ten, quite well.

---

* Provincial Medical Journal, July 30th.

*Case* 2.—A married woman complained of frequent desire to make water; the urine depositing on cooling a whitish sediment; she had been under medical treatment at intervals during the last six months, without deriving any benefit. The benzoic acid mixture was ordered, and she was discharged cured, in three weeks.

*Case* 3.—A man aged fifty had suffered from symptoms of irritability of the bladder for about a month; the urine was acid, but deposited ropy mucus. He took the benzoic acid mixture with great benefit.

In the 4th case, the symptoms being nearly similar, great benefit was derived from the use of the medicine.

The form employed by Mr. Soden is as follows:—

Benzoic acid, one drachm;
Balsam of copaiba, half-an-ounce;

White of egg, enough to form a mixture, with seven ounces of camphor mixture. Two table-spoonfuls to be taken three times a-day.

The medicine deserves a trial.

---

### MIND OR MATTER? THAT IS THE QUESTION!

WE regret to see that the phrenologists, like many people before them, have split into two distinct sects, as the following quotation from Dr. Elliotson's address to the London Phrenological Society, will shew.

" It has appeared to us, that the very first axiom of our science is erroneous. 'The brain is the organ of the mind.'

" Mr. Combe states—'We do not in this life know *mind* as one entity and *body* as another, but we are acquainted only with the compound existence of *mind* and *body*, which act constantly together, and are so intimately connected, that every state of the *mind* involves a corresponding state of certain *corporeal organs*, and every state of *these organs* involves a certain condition of the *mind*.'

" A similar doctrine we shall find inculcated by almost all writers on Cerebral Physiology.

" This is mere assumption. We boast that our science is purely inductive, and yet in the enumeration of our axioms we assume a position all our facts tend to disprove. To evade the charge of materialism, we content ourselves with stating that the immaterial makes use of the material to shew forth its powers. What is the result of this? We have the man of theory and believer in spiritualism, quarrelling with the man of fact and supporter of material doctrines. We have two parties: the one asserting that man possesses a spirit superadded to, but not inherent in brain—added to it, yet having no connexion with it—producing material changes, yet immaterial—destitute of any of the known properties of matter—in fact, an *immaterial something*, which in one word means *nothing*, producing all the Cerebral functions of man, yet not localized, not susceptible of proof: the other party contending that the belief in spiritualism fetters and ties down physiological investigation—that man's intellect is prostrated by the domination of metaphysical speculation—that we have no evidence of the existence of an essence, and that organized matter is all that is requisite to produce the multitudinous manifestations of Human and Brute Cerebration.

" We rank ourselves with the second party, and conceive we must cease speaking of 'the mind,' and discontinue enlisting in our investigations a spiritual essence, the existence of which cannot be proved, but which tends to mystify and perplex a question sufficiently clear, if we confine ourselves to the consideration of organized matter—its forms—its changes—and its aberrations from normal structure."

"We contend that MIND has no existence."*

We were told by a man of no mean note (Berkley) that *matter* had no existence. And now Dr. Elliotson has stepped forward to deny the existence of mind!! Truly, between these two great philosophers, we are likely to be choused out of our existence entirely! Well! never mind—no matter. It is clear that we have neither souls nor bodies—spirit nor substance! We are worse off than the ghosts that crowd CHARON's frail boat—imponderable, intangible, invisible—but still having a *spirit* that escaped from the "organism" above ground!

Does it not occur to Dr. Elliotson that there is a serious responsibility attached to this doctrine? No, he says. TRUTH never can do harm—and VERITAS prevalebit. But is Dr. E. sure that his doctrine is the true one, and that the opinions of all nations and all ages, were the baseless fabrics of visionary superstition?

Granting, *argumenti causa* (what we otherwise deny) that there is no soul—no future state of existence—no rewards or punishments beyond the grave—no truth in natural religion nor in revelation—no difference between a man and a monkey, except a larger head and deficiency of tail—what then? Is the mass of mankind, half of whom, at the least, are plentifully supplied with vicious organs and propensities, prepared for the reception of such doctrines, at the present time, or likely ever to be so? Does he believe that the mere terror of the rope, the dungeon, or the penal settlement, would be sufficient to deter the multitude, or even the enlightened, from crime, if all moral and religious apprehensions were unanimously voted to be bug-bears? We will admit further, for the sake of argument, that human laws and human reason are quite sufficient for the government of society, without any reference to religious obligations. Dr. Elliotson must have seen some hundreds or rather thousands of suffering fellow-creatures, on their beds of sickness and on their death-beds, whose pains were mitigated, whose agonies were soothed, whose fortitude was sustained, and whose dying prospects were illumined by the power of faith, and the hope of immortality!!

> "Unfading HOPE, when life's last embers burn,
> When soul to soul, and dust to dust return,
> What though each spark of earth-born raptures fly
> The quivering lip, pale cheek, and closing eye!
> Bright to the soul thy seraph hands convey
> The morning dream of life's eternal day!"

And would Dr. Elliotson and his band of phrenological MATERIALISTS dash this last cup of enjoyment from the lips of those who have no other stay or consolation on earth, and thus—

> "Hurl the poor mortal trembling from the stage?"

without a ray of hope from the promised blessings of DIVINE REVELATION! For our own parts, were we the most determined Materialists, Deists, or even Atheists, the mines of Golconda would not bribe us to poison the chalice of sorrowing and afflicted humanity in their dying hour, or shake their faith, by engendering doubts in the pious Christian. What right has the cold-blooded MATERIALIST to disturb the creed of Jew or Gentile—of Christian or Mahometan, by thrusting his grovelling and debasing doctrine of annihilation down their throats? The belief in a future state of existence—of rewards and punishments there, even if totally visionary, has an ennobling influence, and pours the balm of consolation daily into the tortured breasts of millions and millions of human beings! Has the SCEPTIC no bowels of compassion on these his fellow-creatures.

---

* Medical Times, July 2, 1842.

### Cases, with Observations on certain Malignant Diseases of the Head and Face. By L. Byron, M.D.*

*Melanosis of the Eye.*

After briefly noticing the discrepancy which prevails amongst pathologists as to the malignancy or non-malignancy of this affection, Dr. Byron proceeds to the relation of several cases.

Case 1. *Medullary Fungus, combined with Melanosis.*—L. Keenahan, æt. 4, of fair complexion, admitted into the Meath Infirmary, January 2, 1826. The right eyeball is much enlarged, so as to form a circular, florid tumor, of the size of a small apple. The upper half is concealed by the eyelid, which is of a dark erysipelatous colour, the lower half is exposed. The tumor is soft, but firm and elastic; on its front is seen the cornea, opaque, disorganized, and superficially ulcerated; with slight muco-purulent discharge. No pain, except when the tumor is pressed upon; natural functions little disturbed. The disease was said to have commenced two months previously, as ordinary ophthalmia; in a fortnight, the ball began to swell, this was accompanied by considerable pain. Vision was lost at an early period; the tumor has enlarged rapidly. His bowels and diet were regulated, and emollient applications made to the tumor. At the end of a fortnight, the health was much improved, but the tumor had increased in size; it was now of an uniform, florid, fungous appearance, with a depression in its centre. Some bleeding occasionally.

On the 20th of January, the eye was removed; very little blood was lost, and the patient bore the operation well.

*Examination of the Tumor.*—The diseased structure presented a good specimen of fungus hæmatodes. It consisted of three lobules; the first, of the size of a pigeon's egg, occupied the seat of the lachrymal gland; the second, of about half the size, lay on the inner side of the orbit; whilst the third and smallest, extended backwards, enveloping the optic nerve and recti muscles, no distinct traces of which were visible. A cavity in the centre was filled by a soft black substance, of the consistence of thick cream, half putrid and very fætid. The sclerotic coat was almost the only vestige remaining of the healthy eye. On cutting into the tumor, its structure was found to be uniformly whitish and semi-cartilaginous. The conjunctiva was thickened, but free from any participation in the disease.

Some fever followed the operation, which was soon subdued. On the 10th day, granulations were seen shooting from the floor of the orbit; and on the 18th the cavity had diminished by one-half. On the fortieth day, however, an unfavourable change took place, he began to look less healthy, and the left eye became amaurotic. On the fifty-eighth day he complained for the first time of pain at the back of the head, and in the right orbit. From about this time the pain in the head increased, the growth of the granulations became arrested, till, on the 74th day, there was excruciating pain at the back of the head. Livid tumor of right lower eyelid; extreme dilatation of pupil of left eye; pulse 120, small and weak; countenance anxious. In this state he was taken home, where he died in a few days. Dr. Byron was not allowed to examine the body.

Case 2. *Melanosis of the Eye, uncombined.—Operation.—Cure.*—This case has already been related in this Journal,* we shall not, therefore, repeat it.

---

* Dublin Journal, July, 1842. † Med.-Chirurg. Rev. No. 72.

Case 3. *Melanosis, combined with Scirrhus of the Globe.*—Rose M'Cabe, æt. 55, of light complexion, admitted August 10th, 1837. About two years ago she discovered a defect in the sight of the left eye. A red fleshy tumor soon presented itself upon the conjunctiva near the cornea, which gradually extended, involving the globe and destroying the sight, which was totally lost about eight months after the disease commenced. There was not much pain till about four months prior to admission, when it became sharp and lancinating, shooting from the eye towards the occiput. On admission, the upper eyelid was tumid and projected, so as to expose about one-third of the swollen globe. The conjunctiva was much chemosed, its secretions apparently healthy. The cornea was opaque and involved in indurated fleshy tubercles, which occupied the whole of the anterior circumference of the globe, and extended as far backwards as the finger could discover. A small portion of the cornea was tolerably clear.

The eye was extirpated on the 20th August. On cutting into the tumor in the upper eyelid, about half an ounce of perfectly black fluid flowed out; the entire of the back part of the orbit was found occupied by the same kind of matter, in which the optic nerve appeared to float. The nerve was not thickened, but near its union with the globe, it was lessened in size, and corroded in two places.

The scirrhous indurations of the globe did not extend so far back as the entrance of the optic nerve. The hyaloid membrane was filled with black fluid, similar to that mentioned above. The lachrymal gland was partially indurated.

The case proceeded favourably, and in a month's time she was discharged well. About two months afterwards, however, she again presented herself, with cancerous ulcerations of skin of the temple, eyelids and cheek, and extended nearly to the bottom of the socket, but there was no return of the melanosis. She returned home, and died about three months afterwards.

Case 4. *Melanosis combined with Scirrhus of the Globe.*—Michael Monaghan, of dark complexion, admitted February 6th, 1838. States that about eighteen months ago, he lost the sight of the right eye; six months afterwards he perceived what he calls a pearl upon the cornea, this has grown very rapidly within the last four months, and is accompanied by intense pain, confined to the eye, temple and forehead. At present the ball protrudes in an irregular tuberculated form, of a dark reddish colour, and discharges dark fœtid ichor which excoriates the cheek. The cornea can be seen imbedded in the disease, and is tolerably clear. The tumor is of about the size of a small orange, firm and unyielding to the touch.

The operation of extirpation was performed, at his anxious desire, on the 12th of February. The pain left his head a few hours after the operation, and in five weeks the orbit had healed; he did not, however, regain flesh or strength. Two months afterwards, he began to complain of cough and pain in the hypochondria, and gradually sunk and died, apparently from exhaustion. On examining the body, five circumscribed black tumors were found on the chest and abdomen, containing a paste-like matter, of a deep brown colour, unorganised, and rendered cohesive by a small portion of fluid. The brain was healthy, as were all the bones forming the walls of the orbit.

*Chest.*—The surface of the lungs was clotted over by a number of melanotic tumors of various sizes, situated immediately under the pleura which formed a partial covering or capsule. The lung around was softened, and more or less black.

*Abdomen.*—The liver presented four large melanotic tumors upon its convex surface, similar in all respects to those in the lungs.

*Remarks.*—The malignant diseases of the eyeball are usually considered to be three in number, viz. cancer, fungus, and melanosis; the two former being distinct; the latter frequently associated with either of the others.

"The first is distinguished as attacking the old; making slow progress; seldom attaining a large size; being accompanied by lancinating pains; and eventually extending to the eyelids and lachrymal glands, and presenting at last a true cancerous ulceration." On being cut into, it is found to be firm and of fibrous or striated texture.

Fungus hæmatodes usually occurs in very early life, and is divisible into three stages. In the first, the pupil is dilated, having at its bottom a peculiar yellow appearance, and the form of the ball is unchanged. In the second stage, the sclerotic is discoloured, and irregular on its surface; the eye appears enlarged, and pain and inflammation have set in. In the third stage, the tumor has forced its way through the sclerotic or cornea, grows with great rapidity, and appears as a dark red fungus, irregular, soft, easily torn, and bleeding readily. On section, it is found to be brownish white, nearly destitute of fibres, and resembling brain in consistency and appearance.

Melanosis of the eye presents few symptoms peculiar to itself, and it is not till it has protruded through the tunics, that the exact nature of the disease can be determined. Then, however, dark livid tubercles show themselves on its surface at different points, soon degenerating into a black fungous mass, which speedily ulcerates, sloughs and bleeds, distending the lids, and forcing its way between them. The discharge is usually thin and of a dark colour. If the disease is uncontrolled, it generally extends to the brain and the patient dies comatose.

On examination after removal, all traces of normal structure are said to be lost in the diseased mass, the greater part of the cut surfaces presenting a deep sooty colour, more or less intermixed with black or brown.

With regard to the propriety of operating in these cases, it would appear that the circumstance of melanotic disease being present, should be no bar, as the fact of melanosis not recurring in any of the cases which have just been recorded, is a proof of its non-malignancy, and of the light estimation in which it may be held as regards an operation.

In extirpating an eye, Dr. Byron thinks that a simple scalpel is all that is required for its performance: the fingers and thumb of the left hand of the operator serving in place of forceps, but, for the division of the optic nerve, where the disease has acquired much bulk, that part of the operation will be best performed by a knife with a lateral curve. Dr. Byron has never found it necessary to transfix the eye to keep it steady during the operation, but what he deems of great importance is, the speedy removal of charpie or lint, where such have been used to restrain bleeding. A piece of lint torn longitudinally, and laid in the wound may be removed easily from eight to twenty-four hours after the operation. Water-dressing with or without light stuffing of oiled lint, as the case demands, is all that should afterwards be used.

*Case of Fungus Hæmatodes of great size, extending within the cranium, without pain, and unaccompanied by Paralysis or disturbance of the Mental Faculties.*

H. O. R. æt. four years and-a-quarter, appeared, about Christmas, 1837, to have taken cold, his breathing was laborious, there was some cough, with discharge of white mucus from the nares; the extremities were cold, the bowels disordered. On the 20th of January, there was slight strabismus of the right eye with drooping of that eyelid; the pulse had become feeble and irregular. On the 25th, the doubtful appearance of his eye was more decided; the upper eyelid covered two-thirds of the ball, and was immoveable, the eye turned upwards and fixed, the iris acting but sluggishly. Sense of smell absent; hearing very acute. Little or no pain in the head.

February 2nd. The sight is suddenly lost; both eyes are fixed and immoveable, with the lids hanging over them. The two front incisor teeth are separated, and a dark-coloured firm elastic tumor has appeared in the line of the palatine suture. The fungoid nature of the disease was now apparent. From this time all the symptoms advanced rapidly, but with regularity; the tumor in the palate protruded outside the mouth to the size of a goose egg; the eyes lost their brilliancy and were converted into two dark-coloured fungi. The nasal and malar bones could be distinctly felt floating wide apart, on this sponge-like mass of disease. His head was thrown back in order to allow of the passage of air; deglutition was performed with tolerable facility, and he could articulate intelligibly. His mental faculties remained perfect till his death, which occurred on the 22nd of March.

*Autopsy.*—The skull-cap adhered firmly to the dura mater. The surface of the hemispheres was vascular, and the arachnoid and subarachnoid tissues had lymph effused in several places. The brain itself was firm, but vascular; some fluid in the ventricles. On raising the anterior lobes of the cerebrum, a firm elastic tumor, covered by dura mater, presented itself, in extent and bulk equal to the segment of a goose-egg, and occupying the space from the anterior edge of the cribriform plate and the foramen magnum. In raising the dura mater, which adhered firmly, a whitish-looking mass, but little elastic, and in some places semi-cartilaginous, was seen; on section, the tumor displayed some black spots or patches of equal density with the rest of the disease.

The sutures between the temporal and malar bones were open on both sides, as were all the sutures of the orbit and face, as well as the coronal suture. No trace of the os planum or os unguis could be discovered. The eye was totally disorganised, some black matter, apparently corresponding with the choroid coat and pigmentum nigrum, was found in the centre of this part of the disease. The seventh pair of nerves passed over the tumor and remained untouched.

The open state of the sutures will explain in some measure, the manner in which the brain eluded the ill-effects of pressure, which would otherwise have occurred from the growth of the diseased mass within the cranium; yet, as the necessity for this change must have occurred before the change took place, it is difficult to account for the very slight functional disturbance which took place.

### *Malignant Osteo-sarcoma of the Lower Jaw-bone; Excision and Disarticulation thereof.*

Anne Lynch, æt. 47, was admitted into the Meath Infirmary, June 2nd, 1828. About twenty years ago she had the last molar tooth on the right side extracted; soon afterwards a small tumor formed in this situation. Twelve years ago, the part was irritated, and from this period the tumor increased, but so slowly as to occasion little inconvenience, up to May last.

At present, the jaw on the right side is considerably enlarged; the soft parts appear healthy, the disease being confined to the bone, from the alveolar process of which has sprung up a fleshy fungous growth, occupying the space from the second molar tooth to about the angle of the jaw.

Caustic was applied to the part, and she was discharged, but returned in the October following, her health being now considerably impaired. The tumor is much increased in size, bleeding freely and giving out a thin fœtid sanies.

On the 6th of October, the diseased mass was removed. After extracting the incisor tooth on the affected side, an incision was made through the soft parts down to the bone in a line with the socket of the extracted tooth, terminating about a quarter of an inch below the chin. The next incision commenced a little anterior to the lobe of the ear, and terminated over the angle of the bone. These two incisions were then united by a third. Having divided the bone in a line with the first incision, the integuments were separated so as to disengage the tumor as far backwards as the angle of the jaw, where the bone was again divided by a chain saw, the bone here consisted only of a thin shell. As the entire of the disease was not included in the detached piece, it became necessary to remove the ramus with the articulating extremity of the bone, which was done with some little difficulty. The divided extremities of the facial and transverse facial arteries were secured; but little blood was lost. The lips of the wound were brought together by sutures. Water-dressing to the wound, and an anodyne draught were ordered. No compression was made upon the carotid artery, nor was it in any way molested.

*Examination of the Disease.*—The bone is enlarged to about the size of a turkey's egg, but of an irregular oblong shape, extending from the first molar tooth to the articulating extremity of the bone. The bone is hollowed out, and is totally absent on the aspect of the alveolar process, which was occupied by the foul excrescences already described. This latter, was continuous with a mass of the disease which occupied about two-thirds of the cavity. Its surface in some places exhibited a very minute vascularity; smooth, but irregular and tuberculated. The part traversed by the chain saw was made up of a brain-like matter, combined with a firmer granular substance, containing various cysts, the walls of which were made up of spiculæ of bone. These cysts contained a firm, white, gelatinous substance, which, however, was easily removed, leaving the walls or internal surfaces smooth, white and irregular.

The case did well, and the subject of the disease is now, eleven years after the operation, in good health.

Dr. Byron draws the following conclusions on the subject of osteo-sarcoma of the lower jaw-bone.

" 1st. The disease almost always commences in the cancellated structure of the bone, and has usually, if not always, a cystic origin, which it maintains, more or less perfectly, throughout its entire growth.

2nd. The disease is always (as far as recorded facts go) mild, under the ages of 28 or 30 years, and although it does not necessarily become malignant after that period of life, still it frequently does so; ultimately involving the soft parts in its neighbourhood, and *uniformly* assuming the character of carcinoma.

3rd. Osteo-sarcoma of the lower jaw-bone is almost always curable by free excision before the soft parts become involved in the disease, which they never do in the benign form of the disease, nor for some time, often several months, after it has changed into carcinoma, or the malignant form.

4th. That cancer of the face, especially the bones, admit of cure more frequently than when seated in any other part of the human frame; cancer scroti perhaps excepted.

5th. Ligature of the carotid artery is unnecessary previous to or during the operation of disarticulation of the jaw-bone.

6th. Stuffing the wound with charpie or lint seems to be, in most cases, unnecessary, and if permitted to remain in the wound beyond eight or ten hours, would most likely excite undue inflammation. No contrivance seems requisite to prevent a retroversion of the tongue upon the pharynx and epiglottis when the bone is divided posterior to the mental attachment of the digastric and anterior fibres of the mylo-hyoidei muscles, as will generally be the case, nor will any contrivance prevent the trifling retraction of the muscles of the face occasioned by their division and want of support."

---

### OBSERVATIONS ON HYPERTROPHY OF THE BRAIN IN CHILDREN.

By CATHCART LEES, M.B.*

Although the much greater development of the brain in some cases than in

* Dublin Journal, September, 1842.

others, as well as its apparent disproportion to its osseous case, has been occasionally noticed by pathologists, yet it is only within the last few years that the attention of the profession, has been directed to whether this condition might not be ranked as a peculiar abnormal state, presenting a group of special symptoms.

The facts hitherto adduced have been such as would tend to excite inquiry rather than produce conviction as to its existence. Though many distinguished authors have slightly alluded to the subject, yet as no points of diagnosis have been laid down to distinguish this state from chronic hydrocephalus, the disease with which it is most likely to be confounded, Dr. Lees has been induced to bring forward some cases which have occurred among the children of the institution to which he is attached, and which may assist in establishing certain data on which to found future observation, as with the exception of Dr. Munchmeyer, of Lunebourg, this peculiar state (as occurring in children) does not appear to have met with the attention which it merits.

*Case* 1.—John Harding, æt. two years, was admitted in May, 1842, for pertussis; his mother states that he has always been healthy, but very *heavy* and *drowsy;* his appetite has always been *great.* His head is rather larger than it ought to be in proportion to his age, particularly across the parietal protuberances; fontanelles perfectly ossified; eyes prominent; intelligence perfect, but appears *apathetic;* the paroxysms of the cough were very violent, and frequently succeeded by convulsions, which were general, and in one of which he died on the sixth day of his disease.

On removing the calvarium, the dura mater appeared to be tense, and on dividing it, the cerebrum appeared swollen, and protruded through the membranes; the convolutions were flattened; the pia mater injected with red blood; substance of the brain, which was very large, firm; there was no effusion in the ventricles; bronchial mucous membrane vascular; a few dark, firm spots, like the clots in pulmonary apoplexy, scattered through the lungs, and quite isolated; the nerves were carefully dissected and presented nothing abnormal.

*Case* 2.—Anne Murphy, æt. 3, a delicate child, with head large in proportion to the body, which is emaciated, particularly the lower extremities; the abdomen is tumid; considerable projection of the frontal bone, as also of the posterior angles of both parietal bones; the eyes are heavy and widely set; the fontanelles closed, but cartilaginous; spends her time in crying, eating, and sleeping. Her intellect is obtuse, but appears to be perfect. Pulse generally regular, pupils natural. Her death took place in some months time, from chronic diarrhœa, without convulsions.

The brain weighed 2lb. 3oz.; the dura mater was firmly adherent to the cranium; the brain was large, the dura mater being rather tense over it; the substance of the brain was firmer and paler than natural; there was no fluid in the ventricles, nor at the base of the brain, in fact, it appeared to be nearly void of either blood or serum.

*Case* 3.—Mary L. æt. 7, delicate complexion, appears as if she is always dropping asleep; pupils natural, pulse regular; her temper is very bad, she will not learn her lessons, but this appears to depend more on indolence than on stupidity; appetite very great; the head is not too large in proportion to the body, but there is considerable projection across the parietal protuberances; complains frequently of headache, and sometimes vomits in the morning.

If we classify the symptoms which the above cases presented, we see that, with regard to intelligence;—

There was a peculiar *obtuseness of intellect,* characterised chiefly by *apathy* to external objects, and a great tendency to drowsiness. There was also evinced a *peculiar irritability of temper.*

The *appetite was very great* in all the three cases, and there existed the *peculiar projection of the parietal protuberances,* on which Dr. Munchmeyer particularly insists, and which may prove a valuable guide in aiding to discriminate this state from chronic hydrocephalus, with which disease it is most frequently confounded. Thus Dr. Hennis Green, in an excellent article on the subject, states that he recently had seen a child who had been condemned to death by a medical man, as having water on the brain, but which was a case of simple hypertrophy, and which did not interfere with the health of the child. The diagnostic sign is, the sense of firmness communicated to the finger on pressure over the fontanelles, in cases of hypertrophy, as contrasting with the fluctuating feel in cases of chronic hydrocephalus; but this could only apply in cases of very young children, or in extreme cases.

The prognosis in children is not necessarily unfavorable, the chief danger, in fact, arises from the occurrence of other diseases, as those attendant on dentition, the exanthemata. The causes of this state are very obscure, but are probably dependent on, or connected with struma; and although the observations which have been hitherto published with regard to this state, bear reference to it as chiefly occuring in adult life, yet we must regard it as the result, either of an abnormal development of the brain, excited before birth, or depending on primary organisation. For as Tiedemann and Valentine have established that the fœtal brain is one of the heaviest and most vascular organs of the body, but presenting little trace of organisation, the primitive type may continue for some years, and thus give rise to hypertrophy, which may remain for a considerable time without interfering with the general health, unless there be either an increase of intensity in its action, or some acute disease supervenes. Thus bearing out the general law of hypertrophy, viz. that the functional disorders which it entails only extend beyond the affected part, according as the part itself extends its spere of action, thus differing from other organic lesions which so soon affect the whole economy, wherever may be the seat of the disease.

---

### OBSERVATIONS ON DISCOLOURATION OF THE SKIN FROM THE INTERNAL USE OF NITRATE OF SILVER, AND ON THE MEANS OF PREVENTING AND REMOVING THAT EFFECT. By CHARLES PATTERSON, M.D.*

Nitrate of silver is undoubtedly a medicine of great service, especially in the treatment of various spasmodic diseases, but the danger of producing discolouration of the skin by its internal administration, prevents its employment as extensively as might otherwise be the case. It must therefore be an object of importance to devise some means of preventing that untoward effect.

Dr. Patterson first quotes the opinions of Dr. A. T. Thomson on the subject, who supposes that the nitrate is taken into the circulation undecomposed, and, arriving in that state at the capillaries of the skin, is there decomposed, and converted into chloride of silver, which is deposited in the rete mucosum. The chloride, he says, acquires a grey, leaden colour from its contact with animal matter; and as it is insoluble, it is incapable of being re-absorbed, is fixed in the rete mucosum, and a permanent stain is given to the skin. Dr. Thomson suggests that, by ordering diluted nitric acid, at the time of administering the salt, its decomposition may be effected.

In opposition to these views of Dr. Thomson's, Dr. Patterson quotes various experiments which he has made, and then brings forward his own conclusions, viz. that the chloride of silver is not the colouring ingredient on which the

---

* Dublin Medical Press, August 24.

blackness of the skin depends; but that the discolouration of the skin is most probably owing to the decomposition of the chloride of silver circulating in the cutaneous tissue through the chemical action of the sun's light, and the deposition there of its metallic basis. All persons are not subject to this accident; for the influence of the sun's rays can only be effective in those cases where the cutis is more than ordinarily vascular and is clothed with a thin transparent cuticle.

The permanence of the stain is not easily accounted for; but it would seem that the metals constitute one class of substances for which the absorbents have no attractive affinity, as is shown in those instances where bullets have remained for years in the body, in the use of metallic ligatures, and in the internal exhibition of quicksilver.

*Means of Prevention—Nitric Acid.*

Dr. Patterson considers that the contemporaneous administration of nitric acid, with the intention of preventing the decomposition of the nitrate of silver, must be entirely useless. The nitric acid undergoes decomposition in its passage through the circulation, and consequently can hardly reach the surface of the body to influence the chemical changes there in operation: and even if it did, and met with nitrate of silver there, its action would be to promote and not to retard the formation of the chloride of that metal; for this reason, that coming into contact with the soluble muriates, it would decompose the muriatic acid, with evolution of free chlorine.

The conclusion to which Dr. Patterson comes on this subject is, that the only way to prevent all risk of discolouration, would be to substitute for the nitrate, some preparation of silver, not liable to be acted on by chlorine, or the sun's light. And happening to be employed in some photographic experiments, his attention was directed to the property displayed by solutions of the iodide of potassium in rendering nitrate of silver insensible to the influence of the sun's rays. When a piece of paper was washed with solution of nitrate of silver, and then immediately immersed for a few seconds in a solution of hydriodate of potash, its colour, even when exposed to the strongest sunshine, remained unaltered. It was evident, in this process, that the hydriodate and the nitrate were both decomposed, and that an ioduret of silver was the result. It then remained to be determined whether, in contact with animal matter or medicinally administered in combination with chemical agents, it would retain that power.

To ascertain this point various experiments were executed;—the ioduret was mixed with different animal and vegetable substances, and submitted to the action of different chemical agents, and then exposed to the action of the sun, without, however, producing the least change of colour.

Having thus satisfied himself as to the chemical habitudes of the ioduret, Dr. Patterson's next endeavours were directed to ascertain its therapeutic effects. The first and principal class of diseases in which opportunities were afforded of administering it, were those various stomach affections to which the Irish peasantry are so very liable, and in which the internal use of nitrate of silver has been found to be most generally successful. They, therefore, afford the best criterion whereby to judge of the comparative efficacy of the ioduret. In such, a number of which Dr. Patterson relates, it proved almost uniformly beneficial. In epilepsy the result was not so satisfactory; but as the medicine was only administered in two cases, it has not had a fair trial in that disease. In hooping-cough it had variable success, but where that complaint was uncomplicated with fever or bronchitis, the ioduret appeared to produce an immediate improvement in the spasms, and hastened the final abatement of the cough. Sufficient time, however, has not yet been afforded, to allow of any definite conclusion to be come to on the subject.

### *Removal of Discolouration of the Skin.*

Dr. Patterson considers that "there can scarcely be a doubt that in those cases, where the skin has become discoloured from the long use of nitrate of silver, the discolouration may be removed by the internal and external employment of suitable preparations of iodine."

The following is the formula which Dr. Patterson employs for the administration of the ioduret of silver.

℞. Iodureti Argenti,

Nitratis Potassæ, āā gr. x.,

Tere simul ut fiat pulvis subtil. dein adde

Pulv. Glycyrrhizæ ℥ss.

Sacchari Albi ℈j.

Mucil. Arab. q. s. M.

Fiant pil. xl. quarum sumat æger j. ter in die.

---

### ON THE USE OF THE IODIDE OF POTASSIUM IN OPHTHALMIC DISEASES, WITH CASES. By ISAAC PARRISH, M.D.*

During a recent term of service at the Wills' Hospital, an opportunity occurred to Dr. Parrish of employing this remedy, the full properties of which are scarcely as yet known in some diseases of the eyes, having a constitutional origin, or closely allied to a scrofulous or cachectic condition of the general system, and the results, he says, though employed in too limited a number of patients to warrant a general conclusion as to its powers, were so striking and satisfactory, as to create a strong impression in its favour.

If this medicine can be safely employed as a substitute for mercury in many diseases of the eye, especially in cases where the state of the constitution renders a resort to mercury hazardous, it will constitute a most valuable auxiliary in the treatment of a numerous class of cases, which are exceedingly difficult to cure.

The dose in which it was administered was from two to six grains, three times daily, in a tablespoonful of the compound syrup of sarsaparilla. The selection of the latter article as the vehicle, constitutes probably an important item in the treatment.

The two following cases are selected, as exhibiting the most decided influence of the remedy under circumstances somewhat embarrassing.

*Case* 1.—William Boyle, æt. 23, was admitted into the Wills' Hospital in the latter part of November, 1841, for granular ophthalmia, from which he had been suffering for four months, and which had been treated on a rigid antiphlogistic plan, with the continued application of emollients to the eyes.

Soon after his admission into the hospital, the inflammation of the conjunctiva became more acute; this was relieved by local depletion from the temples, cooling lotions, &c.; after which the sulphate of copper was applied to the inner surface of the lids; he was placed on a good diet: took syrup of the iodide of iron, &c., under which treatment the eyes rapidly improved. He soon after suffered from another relapse, attended with inflammation and ulceration of the cornea, severe circumorbital pain, with a feeble pulse, and pallid countenance, from which he soon in a great measure recovered. Soon afterwards however he again relapsed, and when he came under the hands of Dr. Parrish, his condition is stated to have been quite deplorable.

---

* Medical Examiner, Philadelphia, No. 16.

He was suffering from deep-seated pain in the head and round the orbit, aggravated at night, and preventing sleep, except under the influence of powerful anodynes; the eyes were highly injected, the sclerotic coat, cornea, and iris being involved. Much intolerance of light and very little vision. The pulse was feeble, skin cool and relaxed, countenance dejected. Some amendment took place from the employment of quinine with the extract of cicuta, counter-irritants to the nape of the neck, anodynes, nutritious diet, &c. This however was of short duration. In the early part of February, erysipelas appeared round the eyelids, rapidly extending over the face, and accompanied with delirium, furred tongue, and feeble pulse. As the eruption declined, the pain in the head diminished, and the condition of the eyes improved, but still the circumorbital pain continued violent, especially at night. A slough was extending on the cornea of the right eye; great intolerance of light, with vascularity of the conjunctiva and cornea continued. The strength of the patient was much reduced; appetite bad, countenance anxious; skin relaxed and pallid; anodynes produced but little benefit; the stomach was irritable and rejected tonics, and fears were entertained that vision would be much impaired, if not lost, by the still active disease of the eyes. It was under these circumstances that the iodide of potassium was resorted to.

An improvement was manifest in less than forty-eight hours, and the pain vanished in a short time, the patient slept soundly without anodynes, strength and appetite rapidly improved, and for the first time since admission he was unaffected by changes of weather.

The application of a solution of the nitrate of silver to the eyes, together with the improvement of the general symptoms, produced a corresponding change in the local inflammation, and when Dr. Parrish left the house, but little doubt existed that he would recover the use of his eyes. He was still taking the remedy in doses of six grains three times daily, and had not suffered from pain in the head since he commenced it.

*Case* 2.—James Dougherty was admitted in October, 1841, for chronic conjunctivitis, which had existed for a considerable time. He complained of a steady dull pain in the side of the head, over the parietal bone, which extended at times over the scalp, and was often very acute. The pain was aggravated at night, was attended with excessive intolerance of light, injection of the conjunctiva, sclerotic and cornea. The patient was greatly affected by changes of weather; much constitutional irritation.

The usual local applications were of no avail; nor did any treatment appear to produce permanent benefit. After trying a great variety of remedies for more than two months, it was at last resolved to administer the iodide of potassium. The change which ensued was surprising. In two or three days the pain was greatly alleviated, so that the patient was able to sleep without anodynes. The appetite increased, and the general health was much improved. The pain disappeared altogether, the injection of the eyes and the intolerance of light were rapidly diminishing, and when Dr. Parrish left the patient under the care of his successor, the prospect of his speedy recovery appeared very encouraging.

*Remarks.*—The most striking benefit which the iodide appeared to produce, was its influence on the severe neuralgic pain, from which the patients had so long suffered. The relief was speedy and permanent, and as a consequence of this immunity from suffering, tranquil sleep was enjoyed, the appetite and strength returned, and the local inflammation subsided.

The remedy was tried in several other cases of strumous inflammation of the eye, in all of which its effect was happy, except in one instance. This was the case of a young woman with scrofulous iritis, in whom the medicine produced severe vomiting and purging, and could not be borne even in two-grain doses.

In the case of a young woman with strumous conjunctivitis, in which the cornea and iris were slightly involved, secondarily, the iodide produced a much more steady and rapid improvement than is usual in this form of disease.

---

### CASE OF PENETRATING WOUND OF BOTH LUNGS.—RECOVERY. By A. N. RUDDOCK, Esq.*

Early in the morning of the 10th May, 1838, a police constable, named Silas Perrott, aged 22, detected a housebreaker robbing some premises which stood in a very retired situation. The policeman attempted to take the man into custody, which he strenuously resisted, and a struggle ensued, during which the latter attempted to throw him into the river, and at last, finding he was likely to be apprehended, drew a long knife with a wooden handle, similar to that used by shoemakers, and gave the constable two desperate stabs in the body.

When seen by Mr. Ruddock, about two hours afterwards, he was tolerably collected, but in a state of extreme exhaustion from the severe nature of the wounds and the consequent loss of blood, which was considerable; much frothy blood was passing from his mouth; he had great difficulty of breathing, with constant cough, and a very small pulse, varying from 140 to 150.

On the right side of the chest was found, on examination, a penetrating wound of rather more than an inch in width, between the seventh and eighth ribs, about four or five inches below, and in a direct line from the axilla; air was passing freely through the wound, and there was considerable emphysema. On the left side there was a corresponding wound, also, between the seventh and eighth ribs, but higher up and more posterior; very little air had escaped into the cellular membrane, but it could be felt crackling underneath a considerable portion of the latissimus dorsi. No doubt could exist as to the lungs being wounded on both sides. As there was no fracture of the ribs, and the wounds were clean, they were closed with adhesive plaster, warmth was applied to the extremities, and some tea administered.

By nine o'clock, a. m., re-action had taken place, and his pulse had become tolerably steady at 120. Venesection to twenty ounces; a mixture of spermaceti and ipecacuanha wine to be taken every three or four hours: fever diet.

At three, p. m., he was labouring under more severe symptoms; his breathing was much oppressed; he had a good deal of pain and uneasiness about his chest, with a full pulse. Venesection to thirty-four ounces, this afforded much relief. At nine in the evening, the symptoms becoming again aggravated, thirty ounces of blood were taken, and some opium administered.

On the following day he was much better, and on the 12th of May, two days after the receipt of the injury, the wounds of the lungs had apparently closed, no air passing through them. From this time he slowly but gradually progressed, and left his bed about three weeks after the injury. The bloody expectoration continued for five or six days, and the emphysema gradually subsided. The wound on the right side of the chest did not heal for a month. Adhesion of the pleura on the right side took place to a considerable extent, covering a space as large as the hand; on the left side, the state of the parts is not so easily ascertained, owing to the thick layer of muscle.

Since that time he has had several attacks of shortness of breath and pain in the side, but they have all given way to a day's confinement in bed, and a mixture of tartar-emetic. These attacks have lately been less frequent.

---

* Provincial Medical Journal, April 2, 1842.

### Case of Tubarian Gestation, with Rupture of the Cyst containing the Embryo, &c. By R. M. Glover, M.D.*

The subject of this case was aged 39, of stout habit; she had been married for the second time for three months. Since her marriage she had not menstruated, though previously she had been regular. During her first marriage she had three miscarriages, but never bore a viable child.

On the 30th of November, 1841, she complained of bearing-down pains in the back and abdomen, with slight bloody discharge from the vagina. On examination, the lips of the uterus were found rather tumefied but close. The pulse was but little affected, the tongue clean. A gentle aperient and anodyne draught were administered and the pains ceased.

In the afternoon of the next day she had a similar attack, when the same treatment was renewed with equal success. This time a few fibrinous shreds were passed with the bloody discharge which was very slight.

About 3 P.M. on the 2nd of December, she was suddenly attacked with excruciating pain in the lower part of the abdomen. When seen by Mr. Dixon, she was in a state of collapse, extremities cold; rigors; lower part of the abdomen extremely painful on pressure; pulse about 80, scarcely perceptible; tongue clean. On examination per vaginam, the lips of the uterus were found less tumid. A draught of laudanum and ether was ordered. Soon afterwards she had severe vomiting, a watery fluid being ejected from the stomach with great force. In the evening, when examined by Dr. Glover in consultation with Mr. Dixon, she remained in much the same state. The abdomen was every where painful on pressure, especially inferiorly, where it had a doughy feel; superiorly, there was some tympanitis. Great pain was especially felt over the iliac fossæ. There was now no discharge from the vagina. The pupils were dilated, and the patient, in a state of partial stupor.

The symptoms of depression continued to augment, she lay during the next day in a state of insensibility, and died at 6 P.M.

*Post-mortem appearances.*—No external sign of pregnancy was observed. On opening the peritoneum, a large clot was perceptible, occupying the space from the umbilicus in front downwards into the pelvis. The intestines exhibited some traces of inflammation. The pelvis and iliac regions and posterior part of the abdomen contained a quantity of dark-coloured fluid blood, estimated at about two pounds, while the clot might weigh three quarters of a pound. No ruptured vessel could be detected, but a sac of the size of a walnut was found where the left Fallopian tube joined the uterus; its interior surface communicated with the peritoneum by an irregular shaggy opening, and by adherent clots, which could be pulled from openings resembling those of veins. The uterus and its appendages were carefully removed and examined subsequently.

The uterus was somewhat enlarged, and its lips slightly tumefied; on being opened, it was found to be lined by the decidua apparently of very recent formation, to within three or four lines of its orifice. This membrane was red, soft and spongy, and terminated towards the mouth of the uterus by a somewhat elevated margin, which may be described as composed of striæ. Air could be injected from the right Fallopian tube into the uterus, but not from that of the left side, nor from the uterus into the cyst already alluded to. The left Fallopian tube was clearly connected with this cyst, although impervious about four lines above it. The cyst has been already sufficiently described.

The left ovary was much shrunk, and a hydatid was observed attached to it. On its outer surface was a recent cicatrix, in which a minute dark clot could be

* London and Edin. Monthly Journal Med. Science, April, 1842.

observed. Near this, on section, two corpora lutea could be observed. The ovarian extremity of the left Fallopian tube was very open, and beautifully fimbriated.

The right ovary displayed two corpora lutea, and several Graafian vesicles.

*Remarks.*—Notwithstanding that the embryo was not found in this case, being probably contained in the huge clot, there can scarcely be a doubt entertained of the nature of the case; and this being granted, the chief remarkable circumstance with regard to it is, the *early period* of gestation at which rupture of the sac took place.

In general, rupture of the sac in case of tubarian pregnancy takes place towards the third month. From the size of the sac in the present case, and the absence of marked symptoms of pregnancy, together with the recent formation of the decidua and state of the uterus, Dr. Glover would be inclined to consider the pregnancy as not more advanced than three weeks. It is by no means improbable that parallel cases are more frequent than is supposed.

---

### Case in which an Aortic Aneurysm burst into the Bronchus.*

On the 8th of February last, Mr. James Goss, second master of H.M.S. Impregnable, then lying in Malta Harbour, struck a man named Edward Purvis on the face. Purvis stooped down, picked up his cap, appeared much excited by the blow, ran up the main ladder, and then again on the upper deck, where he rushed suddenly to a water-bucket and began to drink; immediately afterwards he ejected an immense quantity of blood from the mouth. In five minutes more he was dead, probably about seven minutes from the time the blow was inflicted. Dr. Martin, deputy-inspector, with the surgeons of some of the ships in harbour, were ordered to form a board of inquiry upon the cause of the man's death. The following is the substance of the report of the surgeons, drawn up after the post-mortem examination.

There were no external marks of injury on the body; there was a considerable quantity of frothy mucus tinged with blood exuding from the mouth and nostrils; the external veins were very turgid.

The left lung was greatly engorged with blood, and on removing it from the body, the whole of its substance, together with the air-passages, was found filled with blood. There was some blood extravasated on the right side, but not to the same extent.

There was an aneurysm of the arch of the aorta, where it crosses the windpipe, about the size of a large walnut; the aneurysm had burst into the windpipe.

Absorption of the anterior portions of the bodies of the fourth and fifth vertebræ had taken place. The heart was of the usual size, and apparently healthy. The other viscera also appeared healthy.

On the 17th of February, a court-martial was held on Mr. Goss, when the principal defence of the prisoner was founded on the medical evidence. Dr. M'Arthur declared that "from the appearance of the aneurysmal sac, it must have been on the point of bursting for some time previous to the deceased's death." Dr. Leyson deposed that "the deceased could not, under the circumstances of the disease, have lived any length of time." Dr. Martin stated, that "the blow on the cheek could not have produced the bursting of the aneurysm."

---

* Provinc. Med. Journ. April 16, 1842.

Under these circumstances the court declared the charge "proved in part," and condemned the prisoner to be dismissed from Her Majesty's service.

We are not aware of any case on record precisely similar to the above. There can be very little doubt, but that the excitement produced by the blow occasioned the immediate bursting of the aneurysm, and that any violent emotion would have produced the same effect: but still it is difficult to deny that the blow caused the death.

---

### Pharmacy in Great Britain.

The following is a sketch of the regulations about to be adopted by the Pharmaceutical Society, with regard to those who wish, in future to become members of that body.

Having decided on becoming a pharmaceutical chemist, the student must first be examined in his classical attainments. If residing in or near London, he must present himself for examination at the house of the society, but if living in the country, a certificate of examination by some properly qualified person will be sufficient. At present the Latin language will form the groundwork of the examination, but it is not improbable that at some future period, French, arithmetic, and other branches of knowledge will be included.

The secretary having received the certificate of qualification, the indentures are to be registered, and the apprentice begins his labours. By paying a guinea annually, he is entitled to enjoy whatever educational advantages a connexion with the society can afford.

In order to assume the grade of an associate, he must pass an examination in pharmacy, and this ought to be done as soon as possible after the expiration of his apprenticeship, and in order to remove any unnecessary obstacles to the early attainment of this rank, a *minor examination* has been instituted expressly for associates. The subjects comprised in this examination, are the Pharmacopœia of the London College of Physicians, prescriptions and practical pharmacy. Having passed this examination, he receives a certificate from the board of examiners, stating that he is competent to act as an assistant to a chemist and druggist, and is eligible as an associate of the Pharmaceutical Society.

When the associate wishes to commence business on his own account, his next step is to obtain the diploma of the Pharmaceutical Society, by passing the *major examination.* This comprises the Pharmacopœia of the London College of Physicians; chemistry, materia medica, botany, and pharmacy, as embodied in the Pharmacopœia; prescriptions, and the antidotes for common poisons. The difference between this examination and the former will consist in the degree of proficiency required on all the subjects, and the addition of the science of chemistry, botany, and the rudiments of toxicology; to those enumerated as applicable to associates.

At first, however, the examinations will be somewhat modified, so as to be adapted to the present condition of the candidates, but will be increased in severity as the progress of education allows them to be so, without inflicting injustice on individuals, or injuring the Society, by making the laws respecting admission too prohibitory.—*Pharmaceut. Journal, August.*

---

### Traumatic Tetanus successfully treated by the Sesquicarbonate of Iron. By George Woollam, M.D.*

W. B. æt. 44, a farmer, muscular, and of rather irregular habits, in returning from market on March 29, 1840, fell, when a cart-wheel passed over his right hand. On the same evening he was seen by Dr. Woollam, who found the thumb much contused and lacerated. The case went on very favourably until the 17th of April, when Dr. Woollam, on visiting the patient, found him lying on his left side, in a state of complete emprosthotonos, unable to turn or even to move himself without assistance; deglutition was accomplished with extreme difficulty; the jaws were capable of being separated to such an extent only as to admit of the introduction of a shallow spoon into the mouth. The pulse was 108; pain in the epigastric region; a haggard appearance of countenance; a fixed prominent eye, with occasional profuse perspiration. On examining the wound it was found to be covered with adhesive plaster, and in a very unfavorable condition; a bread poultice was applied to the thumb, and six grains of calomel were given immediately, and in a few hours after, two ounces of castor oil.

Eight in the evening; no alleviation of the symptoms, though the bowels have been freely relieved;—sesquicarbonate of iron, two drachms, to be taken every hour.

April 18, nine, A. M.—No alleviation. The iron to be continued in increased doses. Eight, P. M.: throat feels easier; no evacuation from the bowels since yesterday evening. The castor oil to be repeated as formerly.

April 19, ten, A. M.—Can swallow better; speaks more distinctly; can turn himself in bed, and, when raised, can sit up, though in a bent position. The iron was continued, being exhibited now to the extent of two ounces in two hours, and persevered in till the 27th, when the patient was so far recovered as to be able to walk abroad. From this period the dose was gradually diminished, and, on the 11th of May, entirely discontinued, the disease having completely subsided.

In support of the utility of iron in neuralgic affections, it may be interesting to add, that, a few weeks since, Dr. Shearman, of Rotherham, exhibited it with the most perfect success in a very formidable case of tetanic disease, which had supervened a fortnight previously to his having been called.

---

### Treatment of Encysted Tumors by Creosote.†

Mr. Robinson, of Chapel Street, Grosvenor Square, treats small encysted tumors in this way:—

"Make an incision into the tumor, and discharge its contents; then cleanse out the cyst by means of lint and warm water, and, when thoroughly cleansed, fill the cyst with lint soaked in creosote, which must be removed daily at least, thoroughly cleansing the cyst each day, and inserting fresh lint soaked in creosote. Continuing this treatment for about a week, you will be able, by a pair of dissecting forceps, to remove a part of the cyst, placing the patient in a good light, so that you may be able to see into the cyst when it is dilated. It will most likely happen that the whole of the cyst cannot be removed at once: then the same treatment of cleansing and inserting lint dipped in creo-

---

* Prov. Med. Journ. April 30.

† Med. Gaz. Sept. 2, 1842.

sote must be observed till the whole of the cyst has been removed; and when this has been accomplished, the part will collapse, and scarcely leave any mark."

It is well to be aware that such a method may be practised, although we confess that, in the great majority of cases, we would prefer excision of the cyst. *That* is speedy and effectual, and, taking every thing into the account, perhaps as little painful as Mr. Robinson's more tedious plan. If the cyst is not to be removed by the knife, we should conceive that puncturing it and introducing the nitrate of silver would be more effectual than the creosote. But we have never seen the latter used, and it, is not, therefore, fair to pronounce upon it. Our readers may be induced to give a trial to it, where the patient has a horror of the knife.

---

### Free Trade in Education and Quackery.

The Lancet has some observations upon quackery, which appear to us to be just. But we confess that we do not go the whole way with our contemporary in his free-trade views, nor do we think that science is quite in the same category with hardwares or provisions. The ordinary principles which regulate commerce and barter are either inapplicable to the arts, or demand a considerable modification when they are applied to them.

The Lancet is a free-trader in one direction, but no free-trader in another. In the acquisition of knowledge, it is a free-trader—in its application, a sort of monopolist. It says—" What signifies it where a man gets his knowledge, so that he has his knowledge? but having got that knowledge, and his *degree*, he must be protected."

In accordance with its first position, the Lancet denounces certificates and all that sort of thing. As a corollary of its second, it wars upon quacks.

We are not quite sure that free-trade views are so applicable to *education* as the Lancet supposes.

In the first place, it is by no means very easy to determine, by an examination, what a man does know of a practical art like medicine. He may be at home at question-and-answer work, and, at an examination table, may cut a better figure than one who has really seen more and can do more at the bedside. Every body knows the perfection to which the grinding system has been brought, but there are few, we presume, who argue that gentlemen who acquire their whole professional knowledge in that way, are the best qualified for practice. Any additional guarantee, then, for a really sound education cannot be undesirable. Such a guarantee can only be found in a properly prescribed course of study, the recognition of duly constituted schools.

But this is not the only point of view from which to regard this question. A medical school requires a considerable outlay of capital. To build and keep up dissecting-rooms, stock and maintain a museum, and supply pupils with those conveniences and comforts which are requisite not only for their physical but for their moral health, and last, not least, to induce men of talent and experience to devote their time to teaching, there must be something like a profitable return. It is not to be supposed that men will long work for a barren reputation, or, at all events, that having got what is to be got in that way, they will continue to labour at a loss. The lecturers on the practical parts of medicine ought to be men *in* practice. Are *they* likely to submit to the fag and worry of lecturing without pecuniary remuneration? We apprehend not. Now the free-trade principle applied to schools tends to their indefinite multiplication, nay, to their deterioration. Pupils are practical free-traders too, and they are prone to go where they are *promised* most for the least money. *Grinders* have great charms in their eyes, and, were it left to their own option, to grinders, the bulk of them would hie, and with grinders they would be content. Will any man at all acquainted with medicine contend that this would be a healthy state of things? Will any one maintain that the object of legislation should be to encourage this? We fancy not. Yet undoubtedly the effect of throwing open the school market would be to multiply schools and *grinders,* and in the same ratio to discourage the application of capital, whether monetary or intellectual, on a large scale to teaching.

The celebrated schools upon the Continent which the Lancet so often triumphantly refers to, are not constructed on the free-trade principle. They are found in universities or hospitals, supported by those restrictions and monopolies so odious at home. Nay, it is by virtue of these very monopolies that we get in the German universities a class of teachers comprehending such men as Blumenbach, Meckel, Müller, &c. that cannot be reared in the rough atmosphere of our more *liberal* institutions. Should liberalism take them for its own altogether, hardy plants of the *grinder* sort, would soon be the only ones to flourish.

The majority of the profession, the great mass of general practitioners, are more directly interested in this matter than they may imagine. They are confessedly struggling with difficulties, badly paid, and insufficiently employed. Ask any medical man what he thinks the real evil—ten to one he replies—"the profession is over-stocked." It is almost ludicrous to see in any populous neighbourhood the blue bottles, coloured lamps, and other *affiches* of the faculty. If a new street is built, the first tenant is sure to be a baker, and the next a doctor. Indeed, the latter is often seen, with a courage worthy of a better cause, to emigrate to regions as yet untenanted, and form a sort of pioneer of civilization, to streets and crescents still unborn. The uneasiness and pressure in our ranks is too palpable to be denied. Is such a state of things to be met by measures which would tend to crowd the profession still more? Is a fresh premium, or additional facilities to be offered to men to join us? If the expense of a medical education is at present insufficient to keep down our numbers to a standard consistent with the comforts of the mass, is that education to be made less expensive still? Yet it is of the *expense* of medical education that the Lancet continually complains—the *expense* is what it would diminish. Suppose the economical views of the Lancet acted on. Additional numbers of a lower class, with a lower standard of comfort and respectability, would soon crowd in upon us. Competition would, of course, increase—with that competition the present rate of profits, small as it is, could not be kept up—necessity would drive men to shabby and dishonourable acts—and the result must be to steep the profession in poverty, cover it with discredit, and debase its position in society. The class on which this would principally tell, would be the general practitioner. The aristocracy would always command, and always repay the services of a few physicians and surgeons whose remuneration and place in public estimation would contrast the more strongly with the degradation to which the bulk of the profession would be sunk. In short, we cannot conceive a more injurious or more lamentable state of things than throwing down the barriers which now prevent a rush into our ranks, would give rise to.

One word more, and we have done. The uninitiated might suppose from the denunciations of the Lancet, that the Lecturers, as a class, were fat monopolists, gorged with fees, and accumulating fortunes. To those who know the facts, this is positively laughable. Such are the expenses, that some get nothing, many get next to nothing, most get very little, and very few receive what can be considered an ample recompense. As for the *bulk* of the Lecturers, one has only to look at them to laugh at the idea of *their* being rich. Lean, hungry looking chaps, with high cheek-bones, white complexions, and somewhat rusty coats, they contrast strongly with Mr. Wakley himself, whose coronership, if we may judge from his jolly face, ample waistband, and whole turn out, is worth all the lectureships in London put together.

---

### Citrate and Ammonio-Citrate of Iron.

The citrate of iron has lately been introduced, and is much recommended as a tonic. It is important, however, to observe, that there are two salts similar in appearance, which are occasionally sold indiscriminately as citrate of iron.

The true citrate is very sparingly soluble, and is adapted for pills or powders. The *ammonio-citrate* dissolves with facility, and is a more elegant preparation, when taken in the form of a mixture.

The addition of a few drops of liquor ammoniæ to the citrate, confers solubility on it, by converting it into the ammonio-citrate.—*Pharmaceutical Journal,* April 1.

---

### Artificial Harrowgate Water.

According to Mr. John Mackay, the following is a good substitute for the genuine Harrowgate water:—

℞. Sulphatis potassæ cum sulphure .. ʒj.

Potassæ bitartratis .. .. .. .. .. ʒss.

Magnesiæ sulphatis .. .. .. .. .. ʒvj.

Aquæ distillatæ .. .. .. .. .. .. ℔ij.

Solve. Capiat dimidium pro re natâ.

The above is sufficient for a quart, and ought to be taken early in the morning before breakfast, and be followed by a walk, to produce the desired effect.

The artificial Harrowgate salts are very much employed, and not unfrequently by those who drink the genuine water, for the purpose of increasing its aperient power.

The salts may be made as follows:—

### Harrowgate Salts.

℞. Sulph. potass. cum sulphure .. .. ʒvj.

Potassæ bitartratis .. .. .. .. .. ℥j.

Pulv. magnes. sulph. .. .. .. .. .. ℥vj.

Misce bene.

The usual dose of the above, is one tea-spoonful in a small tumbler-full of tepid water, early in the morning.—*Pharmaceutical Journal,* No. VII.

